# Functional Cyclization of Eukaryotic mRNAs

**DOI:** 10.3390/ijms21051677

**Published:** 2020-02-29

**Authors:** Olga M. Alekhina, Ilya M. Terenin, Sergey E. Dmitriev, Konstantin S. Vassilenko

**Affiliations:** 1 Institute of Protein Research, Russian Academy of Sciences, Pushchino, 142290 Moscow, Russia; olga-alekhina@rcpcm.org; 2 Federal Research and Clinical Center of Physical-Chemical Medicine, Federal Medical Biological Agency, 119435 Moscow, Russia; 3 Belozersky Institute of Physico-Chemical Biology, Lomonosov Moscow State University, 119234 Moscow, Russia; terenin@genebee.msu.ru; 4 Sechenov First Moscow State Medical University, Institute of Molecular Medicine, 119991 Moscow, Russia; 5 Engelhardt Institute of Molecular Biology, Russian Academy of Sciences, 119991 Moscow, Russia; 6 School of Bioengineering and Bioinformatics, Lomonosov Moscow State University, 119234 Moscow, Russia

**Keywords:** eukaryotic mRNA translation, protein synthesis, 5′ cap–poly(A)-tail interaction, polysome, translation reinitiation, ribosome recycling, cell-free system, in vitro translation

## Abstract

The closed-loop model of eukaryotic translation states that mRNA is circularized by a chain of the cap-eIF4E-eIF4G-poly(A)-binding protein (PABP)-poly(A) interactions that brings 5′ and 3′ ends together. This circularization is thought to promote the engagement of terminating ribosomes to a new round of translation at the same mRNA molecule, thus enhancing protein synthesis. Despite the general acceptance and the elegance of the hypothesis, it has never been proved experimentally. Using continuous in situ monitoring of luciferase synthesis in a mammalian in vitro system, we show here that the rate of translation initiation at capped and polyadenylated reporter mRNAs increases after the time required for the first ribosomes to complete mRNA translation. Such acceleration strictly requires the presence of a poly(A)-tail and is abrogated by the addition of poly(A) RNA fragments or m^7^GpppG cap analog to the translation reaction. The optimal functional interaction of mRNA termini requires 5′ untranslated region (UTR) and 3′ UTR of moderate lengths and provides stronger acceleration, thus a longer poly(A)-tail. Besides, we revealed that the inhibitory effect of the dominant negative R362Q mutant of initiation factor eIF4A diminishes in the course of translation reaction, suggesting a relaxed requirement for ATP. Taken together, our results imply that, upon the functional looping of an mRNA, the recycled ribosomes can be recruited to the start codon of the same mRNA molecule in an eIF4A-independent fashion. This non-canonical closed-loop assisted reinitiation (CLAR) mode provides efficient translation of the functionally circularized mRNAs.

## 1. Introduction

More than 50 years ago, electron micrograph images revealed a complex topology of translating polyribosomes. It turned out that both free cytosolic and membrane-attached polysomes commonly form circular arrangements [1,2,3,4]. Although, at the time, the reasons for such polysome topology were not clear, it was posited to enhance mRNA translation via “recycling” of the terminating ribosomes, thus preventing their falling off the mRNA [5]. Later, the so-called closed-loop model for mRNA circularization by means of bridging 5’ and 3´ termini was proposed [6], well before the discovery of the underlying protein interactions [7,8]. Subsequently, the synergy between eukaryotic mRNA 5’ cap and 3’ poly(A) tail in promoting translation was shown [9,10,11], reinforcing the idea that physical interaction between mRNA extremities has functional consequences, i.e., translation enhancement. The direct interaction between the mRNA termini was proved by the discovery of the physical association between the initiation factor eIF4G and the poly(A)-binding protein (PABP) in yeast [12,13], plants [14], and mammals [15]. It was concluded that the proximity of capped 5’ and polyadenilated 3’ ends of mRNA is supported by the chain of RNA–protein and protein–protein interactions—5’ cap/eIF4E/eIF4G/PABP/3’ poly(A). Atomic force microscopy experiments demonstrated the formation of RNA circles when a model capped and polyadenilated RNA was mixed with yeast eIF4F (eIF4E+eIF4G) and PABP, whereas the absence of any of these proteins or their inability to bind eIF4E or PABP prevented RNA looping [13]. Moreover, the formation of eIF4F/PABP-mediated mRNA loops was demonstrated recently in vivo [16]. It is also possible that other protein factors or ribosomes themselves are involved in the formation of an ordered polysome structure bringing mRNA 5′ and 3′ termini together [17,18].

It is worth mentioning that the phenomenon of functional mRNA cyclization is not limited to the classical case of 5’–3’ interactions provided by the canonical cap/eIF4E/eIF4G/PABP/poly(A) bridge. For example, it can also take place on histone-encoding mRNAs that lack a poly(A) tail and possess a conserved 3’ terminal stem-loop (SL), which interacts with cap-associated translation machinery through the SL/SLBP/MIF4G(SLIP1)/eIF3/eIF4F bridge to circularize mRNA and to promote efficient translation [19,20]. Similarly, translation of rotaviral mRNAs that are capped but not polyadenylated is stimulated by their circular topology that is supported by the interaction of the 3’ untranslated region (UTR) with the viral NSP3 protein, which promotes mRNA circularization, displacing PABP from eIF4G [21,22,23]. In the case of picornaviral RNAs that do not possess a 5’ cap structure, internal ribosome entry site (IRES) and poly(A) tail synergistically promote translation, likely by the direct binding of eIF4G to both the IRES and the PABP [24,25,26]. Hepatitis C virus (HCV) RNA carries neither a cap structure nor a poly(A) tail, and, moreover, does not require eIF4G for translation. Nevertheless, it was supposed [25,27] that the HCV RNA is circularized by the chain of protein interactions connecting a specific sequence in the 3’ UTR and the HCV IRES.

Recently, it was reported that methyltransferase METTL3 enhances translation in cancer cells when tethered to mRNAs at m^6^A sites located close to the stop codon. It was shown that METTL3 interacts with the h subunit of initiation factor eIF3 and supports mRNA circularization as a part of the cap/eIF4F/eIF3/METTL3/3′ UTR bridge [28], while m^6^A reader YTHDF1 interacts with eIF3 to facilitate eIF4F-dependent translation [29]. Such mRNA cap-to-tail looping was considered to be necessary for translational control and efficient ribosome recycling during oncogenesis [28]. These examples not only stress the importance of mRNA looping for efficient translation but also imply that peculiarities of specific circularization mechanisms play a secondary role in the translation boost, and the nature of cyclic reinitiation itself seems to be more important.

Establishment of the closed-loop model [6] fueled the belief that the proximity of 5’ and 3’ termini should promote re-utilization of terminating ribosomes (or their small subunits only) at the same mRNA molecule, i.e., should lead to what can be called a functional cyclization of messenger RNAs [30,31,32,33]. Despite the general acceptance of the hypothesis, it has never been proved experimentally, mainly due to difficulties in monitoring the behavior of an individual ribosome. Intense research largely using in vitro translation systems provided ample yet indirect evidence for the closed-loop assisted reinitiation (hereafter abbreviated as CLAR): changes in polysome morphology, mRNA 3’ tail involvement in the initiation process, etc. [18,31,34,35]. The data on synergetic 5’ cap/3’ poly(A)-dependent enhancement of protein synthesis described above, as well as computer modeling [36,37], suggest that it is CLAR that promotes a higher translation level by the increasing of the overall initiation rate. If so, the CLAR-induced boost in the translation product yield directed by a novel mRNA molecule will show up not immediately but rather after at least two rounds of translation, when the first pioneer ribosome(s) enter the second termination/recycling stage. In an in vitro translation experiment where all exogenous mRNA molecules are obviously ribosome-free at the start of the reaction, this should be reflected in a deferred increment in protein synthesis rate. Such peculiarity of translation kinetics would be strong indirect experimental evidence for the CLAR. 

Implementation of such analysis has been hampered in routinely used protein yield assays, as subtleties of kinetic curves were obscured by the large statistical spread of data points. To circumvent these limitations, we used the advantages of an alternative method—in situ monitoring of protein synthesis. Real-time monitoring of the translation of a luciferase mRNA was performed by continuous in situ measurement of the luminescence in the translation mixture. The resulting smooth kinetic curves had a high signal-to-noise ratio and thus were suitable for detailed numerical analyses. The power of the method was previously demonstrated in the studies of cotranslational protein folding [38], changing of translation rate in the course of polysome formation [39] and the temporal parameters of ribosomal scanning [40].

Here, with the use of this technique, we succeeded in detecting the aforementioned increase of initiation rate in the course of translation in a mammalian cell-free system. Sedimentation analysis of translating polyribosomes indicated that this acceleration reflected the increase of initiation rate. The observed effects strongly depend on the interaction between 5’ cap and 3’ poly(A). We determined the optimal lengths of mRNA 5′ UTR and 3′ UTR, in terms of the highest translation acceleration level, as ~80 and ~300 nt, respectively, which is close to the common lengths of mammalian UTRs. The reinitiation events responsible for the increase of translation rate were shown to be quite distinct from the classical initiation mechanism, as they had a relaxed dependence on the canonical initiation factor eIF4A. All the collected data show that the circularization of mRNA switches on the closed-loop assisted reinitiation of translating ribosomes that utilizes the alternative initiation mechanism.

## 2. Results

### 2.1. Biphasic Kinetics of Capped and Polyadenylated mRNA Translation

Previously, it was shown that the introduction of luciferin to the translation mixture allows measurement of the activity of firefly luciferase (Fluc) directly in the reaction tube [38,39]. The continuous measurement of luciferase activity provides a time course of translation as a smooth kinetic curve with a high signal-to-noise ratio. Since folding of the firefly luciferase occurs co-translationally, the enzyme exhibits its activity immediately upon translation termination [38]. The kinetic data thus can be interpreted as an in situ monitoring of the accumulation of newly synthesized luciferase. A significant delay in signal appearance reflects the obvious fact that the first full-length (i.e., active) luciferase molecules cannot appear in the reaction mixture until the first ribosomes finish translating the mRNA. Therefore, the time lag between the start of the reaction and the appearance of a full-length, active luciferase can be used to estimate the time required for the synthesis of a complete protein molecule during a full round of translation consisting of initiation (including scanning), elongation, and termination. It was shown that, in the case of mRNAs with leaders of a moderate length, this lag nicely corresponds to “transit time” (elongation + termination), the more common measure of the duration of translation [39].

**Figure 1:**
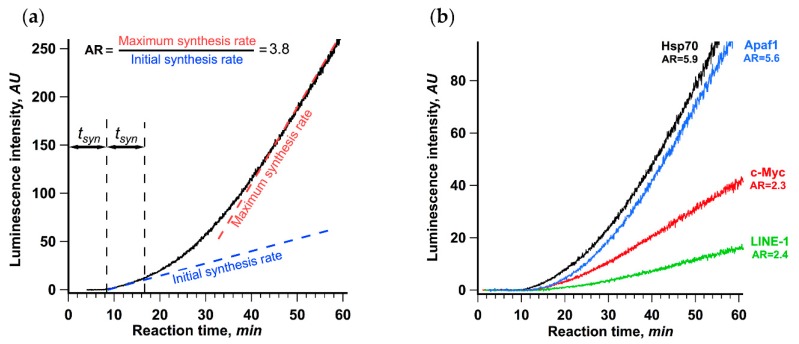
Rate of protein synthesis increases in the course of translation of capped and polyadenylated mRNA, as revealed by continuous in situ measurement of luminescence. (**a**) βgloFlucA50 mRNA (25 nM) was translated in a Krebs-2 cell-free translation system at 30 °C. The dashed lines depict the initial synthesis rate as the slope of the linear fit of the data collected during the first 5 min after the appearance of active product and the maximum synthesis rate as the maximum slope of the kinetic curve achieved in the course of translation reaction. The ratio of the maximum and the initial synthesis rates was used as a measure of the translation acceleration (AR, stands for “acceleration rate”). The initial translation rate was determined as a linear approximation of a 5 min fragment of a kinetic curve right after appearance of luciferase activity; maximum translation rate was determined as a maximal value of the slope of linear approximations obtained for a 5 min window sliding along the whole kinetic curve with 2.5 s step. (**b**) Four Fluc encoding mRNAs with 5′ untranslated regions (UTRs) of different human genes (HSPA1A, 5′ UTR is 219 nt long; MYC, 421 nt; APAF1, 580 nt; and LINE-1, 908 nt) were translated in the same system. In both cases, the representative curves out of at least three replicates are shown.

Figure 1a shows the result obtained for the translation of βgloFlucA50 luciferase mRNA possessing 5′ UTR of rabbit β-globin mRNA, the SV40 3′ UTR and A_50_ sequence at the 3’ end. The most noticeable feature of the data is the clear biphasic nature of the curve showing the accumulation of active luciferase over time. The rate of the protein synthesis started to increase after 18 min of translation reaction, which is roughly equal to two lag periods, or twice the transit time. We defined the initial synthesis rate as the average luminescence increase rate during the first 5 min after the detection of the first active product. The maximum rate was defined as the maximum luminescence increase rate, or the maximum slope of the kinetic curve, achieved in the course of translation reaction (see Materials and Methods section for the detailed description). The ratio of the maximum and the initial synthesis rates was taken as a measure of protein synthesis acceleration. For the translation of βgloFlucA50, this value was equal to 3.8, which manifested as a visible kink in the kinetic curve. 

We also studied translation of four Fluc encoding mRNAs with 5′ UTRs of various lengths derived from human cellular mRNAs (Figure 1b), which all direct translation in a 5′ end and scanning-dependent fashion, while having different dependence on the 5′ cap [41,42]. All of them demonstrated a significant increase in the protein synthesis rate in the course of translation reaction with the acceleration rate varying from 2.3 to 5.9. This result suggests that the observed acceleration is a general phenomenon of cellular mRNAs translation and may be an important feature of eukaryotic translation initiation. Interestingly, the translation of mRNAs with the Hsp70 and the Apaf-1 leaders, which was reported to have a relaxed cap dependence but still employed the 5′ end dependent scanning (for review, see [43,44,45]), revealed the highest acceleration rates. In contrast, translation of two virus IRES-containing uncapped polyadenylated transcripts with no scanning and, apparently, no circularization involved, PTV-Fluc and CrPV-Fluc [46,47] (with the IRESs from porcine teschovirus and cricket paralysis virus, respectively), showed no acceleration of translation under the same conditions (Supplementary Figure S1).

### 2.2. The Acceleration is not Caused by Involvement of New mRNAs in Translation but Rather Reflects an Increase of the Initiation Rate

Assuming the rate of elongation to be constant, the acceleration of protein synthesis can be explained either by an increase of the initiation rate or by involvement of new mRNA molecules into the translation process. The bare analysis of the time-course curves did not allow us to distinguish between these two possibilities. To resolve this issue, we analyzed a change of polysome distribution profile in the course of translation. There is a simple test—if the elongation rate is constant, the continued increase of the number of ribosomes per mRNA (“heavy shift” of the polysomes) after the first rounds of translation indicates the increase of initiation rate (see, e.g., [48]).

The whole-cell Krebs-2 extracts we used contain a significant amount of intact endogenous mRNA; therefore, the conventional UV detection of polyribosomes formed solely at certain mRNA was impossible. We thus used the ^32^P-labeled mRNA sample; therefore, the radioactivity of fractions measured along the polysome profile reflected the distribution of polysomes formed on the studied mRNA.

**Figure 2:**
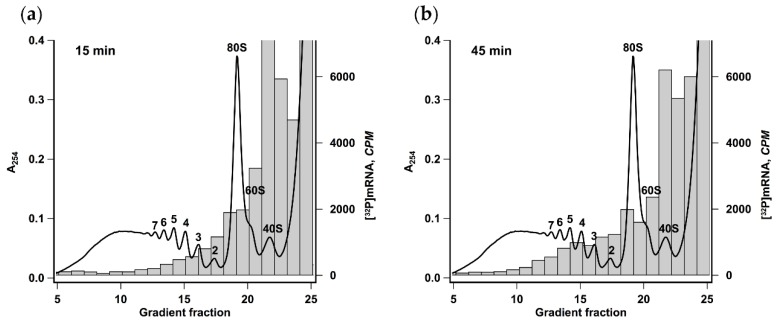
Size of polyribosomes increases in the course of translation. Krebs-2 system translating [^32^P] βgloFlucA50 mRNA was fractionated by centrifugation in 12 mL 15–45% sucrose gradient after 15 min (**a**) or 45 min (**b**) of translation reaction. Radioactivity of 0.5 mL fractions was measured by Cherenkov counting. The absorbance curve representing polysome distribution in HEK293T cell lysate fractionated under the same conditions is given as a reference. Numbers indicate the size of polyribosomes in the corresponding peak.

Polysomes formed after 15 or 45 min of translation reaction were analyzed by ultracentrifugation in a linear sucrose gradient (Figure 2). It was seen that there was a pronounced heavy shift in radioactive mRNA distribution reflecting the increase of the average polysome size from two to five. This result implies that the observed acceleration was caused not by employing the new mRNA molecules but by the increase of initiation rate or the frequency of ribosomes recruitment by each mRNA molecule.

Therefore, one can conclude that, after the first round of translation, the overall initiation rate increased, which was manifested as a deferred protein production boost once the newly reinitiated ribosomes accomplished the second round of translation.

### 2.3. Integrity of Cap-to-Tail Interactions is Necessary for the Acceleration of Protein Synthesis

The obtained data suggest that the ribosomes that complete translation can somehow boost the initiation process. It is reasonable to assume that the terminating ribosome can restart translation at the 5’ end of the same mRNA molecule, providing “cyclic” translation. If so, the acceleration should be very sensitive to the integrity of the chain of interactions [cap/eIF4/PABP/poly(A)] that bridges mRNA termini together. 

**Figure 3:**
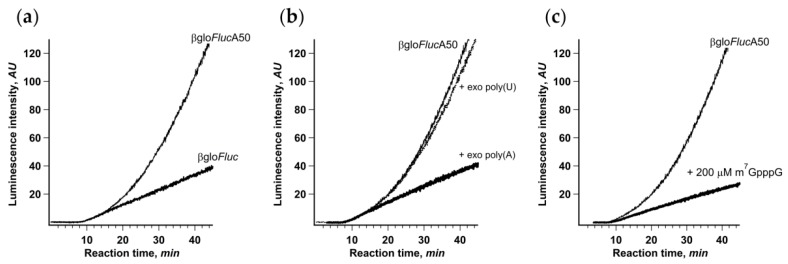
Integrity of mRNA cap-to-tail interactions is crucial for the acceleration of translation. (**a**) Continuous in situ monitoring of the translation of mRNA with (βgloFlucA50, thin line) and without (βgloFluc, bold line) 3’-A50 sequence in a Krebs-2 cell-free system; (**b**) addition of high-molecular weight exogenous poly(A) (to 25 μM of adenylic residues) to the Krebs-2 cell-free system translating βgloFlucA50 mRNA; (**c**) addition of cap analog (m^7^GpppG) to the same translation system.

The easiest way to break these cap-to-tail interactions is to use mRNA lacking either 3’ end poly(A) sequence or m^7^G-cap. As noncapped mRNAs are not translated in Krebs-2 extract efficiently enough to produce high-resolution kinetic curves, we opted for translation of a non-polyadenylated βgloFluc transcript with the same 3′ UTR (Figure 3a). In this case, the kinetic curve had the ordinary one-phase cumulative shape and did not reveal any acceleration effect. On the other hand, the initial translation rate was almost the same as in the case of βgloFlucA50, implying that the first acts of initiation were not affected by the presence of a poly(A) tail. 

As an alternative, we added high molecular weight polyadenylic acid to the translation system in trans, as a competitor for the PABP molecules bound to mRNA poly(A)-tail in cis. Polyadenylic acid is a well-known inhibitor of translation, but here, we demonstrated that its inhibitory effect is almost entirely due to the suppression of the acceleration effect, since the initial translation rate remained virtually unchanged (Figure 3b). This is also a good example of how the results could be under-interpreted if the important data are hidden behind a crude method.

We also used the cap analog m^7^GpppG as a specific competitive inhibitor of cap–eIF4F interaction (Figure 3c). It is known, though, that in sub-millimolar concentrations, cap analog has a limited effect on cap-dependent translation in vitro, most probably due to much lower affinity to eIF4F compared to capped mRNA 5’ end. Indeed, the addition of 200 μM m^7^GpppG to the system translating βgloFlucA50 had only a limited effect on the initial translation rate. At the same time, it completely eliminated the acceleration of protein synthesis. The plausible explanation would be that, as a competitive inhibitor, m^7^GpppG is in equilibrium with capped 5’ end in eIF4F binding, which is enough for occasional de novo cap-dependent initiation but prevents maintaining permanent mRNA termini interaction necessary for circularization. All these results imply that the stable physical interaction between mRNA termini is necessary for the acceleration effect observed.

### 2.4. Acceleration Rate Depends on the Length of 5’ and 3′ UTRs

In the framework of the CLAR model, the mutual arrangement of stop and AUG codons is very important. It is evident that the spatial distance between the point of mRNA termini interaction and the codons where the acts of termination and initiation take place should play a prominent role here. Changing the lengths of 5′ UTR and stop-to-poly(A) link is the easiest way to vary this distance, although not unambiguously.

We performed translation of βgloFlucA50 along with other mRNA constructs, possessing various fragments of anomalously long human retrotransposon LINE-1 mRNA leader as 5′ UTRs. Initiation at these reporter mRNAs was previously shown to be strictly cap-dependent [49], and the initiation time at the beginning of translation reaction linearly depended on their length, implying a canonical scanning-dependent initiation mechanism [40].

**Figure 4:**
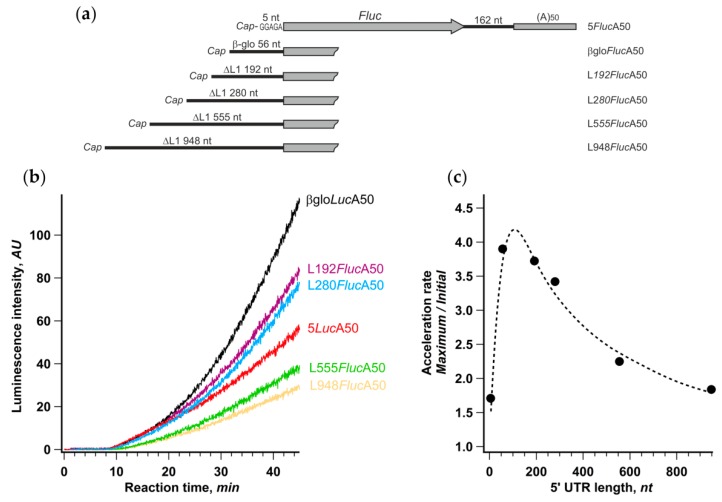
Degree of acceleration depends on the length of 5′ UTR. (**a**) Schematic representation of mRNA constructs with different lengths of 5′ UTR; (**b**) time course of cell-free protein synthesis directed by the indicated mRNAs; (**c**) dependence of translation acceleration rate on the length of 5′ UTR. The values of the acceleration rate were calculated as described in the legend to Figure 1.

Despite the significant, up to fivefold, differences in the final protein yield, the initial translation rates were basically the same in the case of mRNAs with the 5′ UTR lengths ranging from 5 to 280 nt and dropped only twofold in the case of constructs with longer leaders (534 and 928 nt). Therefore, the overall efficiency of translation in each case was dictated mainly by the rate of acceleration that varied from 1.7 to 3.9 (Figure 4c). The acceleration rate dependence on the 5′ UTR length was distinctly bell-shaped with the maximum around 80 nt, which falls well within a size range for highly expressed mammalian mRNAs’ leaders (for review, see [50]). Thus, the support of efficient CLAR is conceivably one of the factors that determine the optimal length of mRNA leader.

It should be noted that the increase of the 5′ UTR size brought additional RNA structures that moderately affect the initiation efficiency [40] but could also impact the acceleration rate. It is also important that mRNAs utilizing non-conventional translation initiation pathways, such as those having the APAF1 and the HSPsp70 5′ UTRs (Figure 1b), or directed by virus IRESs (Supplementary Figure S1), may fall out of this dependence, while the c-Myc 5′ UTR containing reporter almost perfectly fits it (421 nt long 5′ UTR, AR = 2.3).

Interestingly, translation of an mRNA with a very short leader (5 nt) revealed the weakest acceleration effect. This suggests, in the context of the cyclic reinitiation model, that the distance between the cyclization point (cap) and AUG can be too small for CLAR to occur efficiently while still being enough for the efficient de novo cap-dependent initiation (Figure 4b, see also [51]).

**Figure 5:**
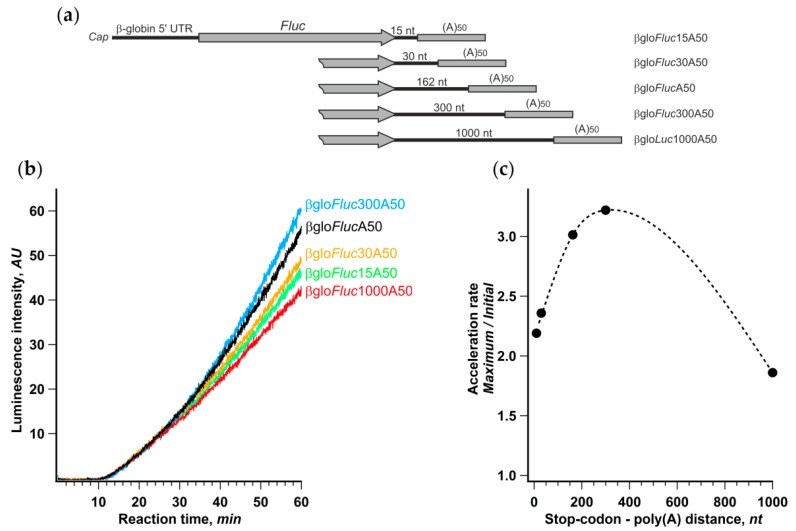
Distance between stop-codon and poly(**a**) tail affects the rate of acceleration. (**a**) mRNA constructs with varying length of 3′ UTR; (**b**) kinetic curves of the translation of indicated mRNAs in a Krebs-2 system; (**c**) dependence of translation acceleration rate on the stop-to-poly(a) distance.

Further, we changed the distance between the termination codon and the 3’ poly(A) by reducing the length of the 3′ UTR in βgloFlucA50 construct or, conversely, by adding vector fragments to it (Figure 5). The translation of these transcripts in the Krebs-2 cell-free system revealed a very weak dependence of the initial translation rate on the 3′ UTR length. At the same time, the noticeable difference in the acceleration rates led to 30% variation of the final yield of luciferase activity. The acceleration dependence on the stop-poly(A) linker length had a pronounced maximum around 300 nt that was fairly close to the mean distance between the stop codons and the closest downstream poly(A) sites in human and mouse genes [52]. Although certain genes have very diverse 3′ UTR lengths (for review, see [35]), this could be regarded as additional evidence in favor of an assumption that common characteristics of cellular mRNAs may have something to do with the observed phenomenon.

**Figure 6:**
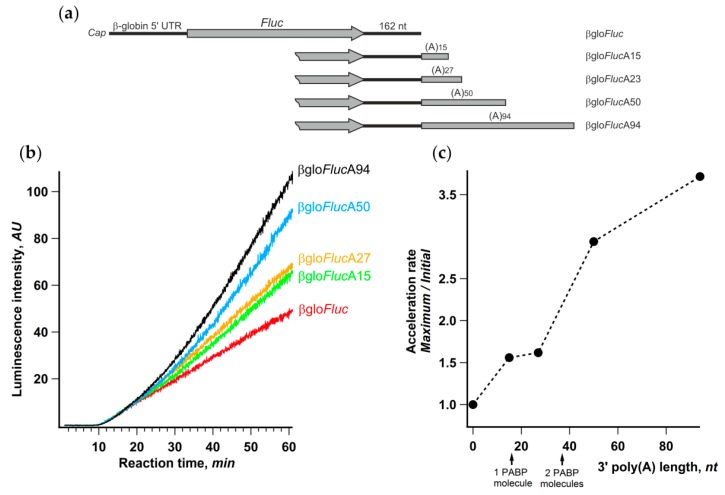
Extension of the 3’-poly(**a**) sequence improves the acceleration effect. (**a**) mRNA constructs with the gradually increasing length of the 3’-poly(A) sequence; (**b**) kinetic curves of the translation of indicated mRNAs in a Krebs-2 system; (**c**) effect of the length of 3’-poly(A) on the translation acceleration rate. The lengths of minimal poly(A) sequences that can bind one or two poly(A)-binding protein (PABP) molecules are indicated by arrows.

It is well known that, in living cells, the length of mRNA poly(A)-tails is dynamic and varies significantly for different transcripts [53,54]. Therefore, it seemed interesting to assess the relationship between the length of the reporter mRNA poly(A)-tail and the acceleration effect. We prepared a set of βgloFluc mRNAs either possessing 15, 27, 50, or 94 nt long poly(A)-tail or lacking any (Figure 6a) and analyzed their translation in the Krebs-2 cell-free system. In accordance with numerous published observations (e.g., [55]), the luciferase yield after 1 h of translation substantially increased with the poly(A) lengthening (Figure 6b). However, the luciferase synthesis rates at the initial phase of translation reaction were the same for all five transcripts, while the difference became evident at a time point that roughly corresponded to two transit time periods (Figure 6b). Accordingly, the ratio between maximal and initial luciferase accumulation rates clearly increased with the extension of the poly(A) tail (Figure 6c). Little difference in the acceleration rates for 15- and 27-nt long poly(A) can be explained by the fact that, while PABP binding requires poly(A) sequence of as few as 12 nt long, it covers at least 27 nt RNA fragment (reviewed in [56]).

### 2.5. eIF4A Dependence of Initiation Decreases in the Course of Translation

ATP-dependent DEAD-box helicase eIF4A is an important component of the translation initiation machinery, a part of eIF4F protein that provides 43S preinitiation complex attachment to an mRNA 5′ end and subsequent 5′ UTR scanning. Dominant negative R362Q mutant of eIF4A is a well-studied inhibitor of translation initiation [57]. Due to the lack of ATP binding ability, it cannot bind and unwind RNA [58], while its affinity to eIF4G remains unaffected by the mutation. Sensitivity to eIF4A(R362Q) inhibition is clearly an indication of ATP- and scanning-dependent initiation of translation [57,59].

**Figure 7:**
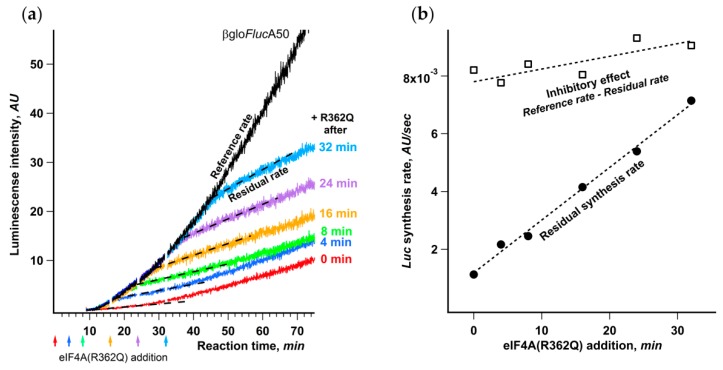
Dependence of the initiation on eIF4A decreases in the course of translation. (**a**) eIF4A(R362Q) was added to final concentration of 40 μg/mL to the same reaction mixtures of Krebs-2 system translating βgloFlucA50 mRNA at the time points indicated by arrows. The dashed lines represent the residual synthesis rate as the slope of the linear fit of the data collected during the 5 min after the occurrence of eIF4A(R362Q) inhibitory effect; (**b**) dependence on the time eIF4A(R362Q) addition of the residual synthesis rate (●) and the inhibitory effect (□) as the difference between the residual synthesis rate and a reference synthesis rate, where the latter is a slope of the kinetic curve of uninhibited reaction determined at the same time range as the corresponding residual synthesis rate.

The addition of eIF4A(R362Q) to Krebs-2 cell-free system translating βgloFlucA50 to the amount approximately equimolar to endogenous eIF4A led to almost complete abrogation of translation (Supplementary Figure S2). To check whether the effect of eIF4A(R362Q) changes in the course of translation reaction, we added an equal amount of the inhibitor at different time points (Figure 7a).

The uniform delay of inhibitory effect in response to the mutant addition that coincided well with the initial lag in luciferase appearance makes it apparent that the ribosomes that had already started translation retained the steady rate of protein synthesis. This is an independent proof that eIF4A(R362Q) affects strictly the initiation stage and has virtually no influence on the rates of elongation and termination.

The most interesting conclusion that can be made from the analysis of the kinetic curves is that the inhibitory effect of eIF4A(R362Q) on the initiation markedly depends on the moment of its addition to the reaction mixture. The later the inhibitor is added, the higher is the residual luciferase synthesis rate after the inhibition showed up (Figure 7b). At the same time, the inhibition level, determined as the drop in protein synthesis rate, remains virtually constant regardless of the moment of eIF4A(R362Q) addition. 

Apparently, there is some fraction in the multitude of initiation events that increases in the course of translation reaction and is weakly affected by eIF4A(R362Q). The most straightforward explanation is that two quite different and weakly interfering initiation mechanisms coexist. First one is de novo ribosome recruitment through the canonical cap-dependent initiation that involves cap recognition and scanning stages, which are highly responsive to the inhibition by eIF4A(R362Q). The second one is presumably a non-canonical initiation mechanism that is weakly sensitive to eIF4A(R362Q) and only manifests itself after an mRNA chain becomes loaded with translating ribosomes. CLAR can be considered as a likely candidate for this alternative initiation pathway. In the case of a moderate de novo initiation rate, when the ribosome density along the coding region is not very high, these two initiation mechanisms do not have to compete vigorously for mRNA 5’ end and/or AUG codon and can act independently of each other, which should be manifested as an increase in the overall initiation rate.

## 3. Discussion

Translational control by mRNA poly(A)-tail, mediated by formation of the closed-loop structure, is a widely accepted fact in molecular biology inspiring researchers for decades [53,54,60]. However, even general principles of this regulation are still not well understood. In particular, the stimulatory effect of the poly(A)-PABP complex on translation can be explained either by direct enhancement of de novo initiation (through stabilization of initiation complex by modulating eIF4F, eIF4B, 40S, or 60S binding to the mRNA 5′ termini [12,61,62,63,64,65,66,67]) or by the recruitment of ribosomes, which terminated at the stop-codon of the same transcript, i.e., by CLAR, as suggested by the closed-loop model [6]. The latter explanation is supported by well documented evidence of functional mRNA cyclization in the absence of the canonical cap/eIF4E/eIF4G/PABP/poly(A) bridge, e.g., in the cases of non-polyadenylated rotaviral and histone mRNAs, or IRES-containing picornavirus and flavivirus genomic mRNAs (for review, see [33,68]). 

However, the versatility of the mRNA circularization has been challenged recently by the indications that the closed-loop conformation is rarely observed in living mammalian cells under normal conditions [69,70], that the predominant form of actively translated mRNA is likely not circularized in yeast and mammals [16,71], and that the polysome topology in a plant cell-free system undergoes complicated step-wise evolution accompanied by translation efficiency changes [72,73]. On the other hand, new evidence of functional mRNA cyclization came from recent studies of m^6^A mRNA methylation that brings 5′ and 3′ UTRs together via eIF3h–METTL3 interaction and/or cap/eIF4F/eIF3/YTHDF1/m6A bridge formation, which facilitates translation during oncogenesis [28,29]. These new data require a revision of the closed-loop model by focusing at the mRNP structure dynamics during its entry into polysomes [32,74].

Employing the precise in situ analysis of the time course of cell-free protein synthesis, we show here that cap-to-tail looping of eukaryotic mRNA does not enhance de novo initiation of translation. It turns out that only after the first ribosome accomplishes the translation cycle does the looping start to positively affect the initiation rate (Figure 1). Notably, the acceleration of translation is accompanied by the loading of the reporter mRNA with additional ribosomes, while the rate of elongation remains constant, which proves that it is the increase of total initiation rate that is responsible for the stimulation of translation (Figure 2). We also show that the acceleration depends on the protein bridge connecting the capped 5’ end and the 3’ poly(A)-tail of eukaryotic mRNA and is proportional to the poly(A)-tail length (Figure 3, Figure 6). We observed no acceleration in the case of the PTV or the CrPV IRES-dependent translation, which is not eIF4F-dependent [46,47] and most likely cannot be enhanced by the mRNA circularization (Supplementary Figure S1). It is entirely possible that these results reflect the in cis reinitiation of translating ribosomes, in other words, cyclic mRNA translation, a phenomenon that is generally accepted but has never been proved experimentally.

Our results reveal that the distance of ~300 nt between stop codon and poly(A)-tail is optimal for the CLAR (Figure 5). At first glance, such a substantial distance seems rather confusing. It is obvious that efficient cyclic reinitiation of translation requires close proximity of recycled ribosomal subunits and 5’ end of the translated mRNA. Considering that it is the poly(A)–PABP interaction that provides physical connection of mRNA ends, the distance between stop-codon and poly(A) tail is likely rather short. It should be noted, though, that the distance between stop codon and the 3’ poly(A) may not directly depend on the length of the interconnecting mRNA sequence. First of all, an almost inevitable presence of secondary structure elements pulls together the ends of any sufficiently long RNA fragment [75]. Besides, it was shown that termination factor eRF3 serves as a bridge between termination/post-termination complex and PABP through specific protein–protein interactions [76]. It is reasonable to assume that formation of such a linkage between two sites on mRNA requires certain length and flexibility of the interconnecting nucleotide chain. Thus, the stop-codon/poly(A) RNA linker should not be very short; rather, its size should allow a formation of the RS/eRF3/PABP/eIF4F/5’ cap protein bridge necessary for CLAR. In this regard, it is also notable that longer poly(A)-tails provide stronger translation acceleration (Figure 5), suggesting the importance of more than one PABP molecule bound to the transcript, which allows their multiple simultaneous interactions with other translation components.

It is still not clear how mRNA looping promotes reinitiation by means of the recycled ribosomes. The simplest possible mechanism suggests that bridging the ends of mRNA increases local concentration of recycled ribosomal subunits in the vicinity of the mRNA region of ribosome recruitment, i.e., mRNA 5′ end. Such an explanation is certainly plausible, but some experimental data, including those presented herein, suggest that there is a special mechanism that facilitates CLAR.

Probably the least expected observation made here is the partial resistance of CLAR to the inhibition by eIF4A dominant-negative mutant R362Q (Figure 7). This protein is the well-known inhibitor of ATP-dependent eIF4F/eIF4A-mediated ribosomal scanning. It drastically impairs not only canonical cap-dependent initiation but also those types of IRES-dependent initiation that involve scanning stage to reach a start codon [57]. However, translation of mRNAs with a relaxed dependence on eIF4F/eIF4A has been shown to be insensitive or partially resistant to this inhibitor [59,77,78,79]. In our experiment, translation of the βgloFlucA50 mRNA directed by the classical cap-dependent leader is indeed inhibited. However, the later R362Q is added, the higher residual translation rate is observed (Figure 7). This means that, at the later stages, a fraction of initiating ribosomes reaches the start codon in an eIF4A/eIF4F- and likely ATP-independent manner. We suggest that this fraction corresponds to the ribosomes employing CLAR, and this pathway likely does not involve regular scanning.

Although the mechanism of CLAR is out of the scope of this study, we can propose some mechanistic insights into this phenomenon. First of all, unlike regular initiation, CLAR could be facilitated by some protein factors that remain bound to the reinitiating 40S subunits after the previous (termination or ribosome recycling) steps. This could be ABCE1, a ribosome recycling factor known to participate also at the initiation step (reviewed in [80]), or eIF3, which may be recruited to the termination complex and is known to facilitate at least the regular (in cis) reinitiation (for review, see [81]), or even PABP, which is directly involved in termination (see [82] and references therein), while it also affects eIF4F and eIF4B function at the 5′ end (see above). An intriguing possibility is the involvement of recycling/reinitiation factors eIF2D and MCT-1/DENR in CLAR [83,84]. Thus, the reinitiating ribosomes, in contrast to “naïve” (de novo entering) ribosomes, can be already loaded with components necessary for 5’ UTR binding, scanning, and start codon selection, providing the relaxed dependence on the scanning factors such as eIF4F/eIF4A.

The advantage of CLAR ribosomes may also be their conformation. To leave the mRNA chain, the recycled 40S subunit has to acquire an “open” conformation, which could be preferential for 5’ UTR binding and AUG selection. Whether this conformational switch can be assisted by the 40S binding capacity of PABP [61] or other translation factors located nearby is unknown. If the mRNA chain is organized in a way providing a close proximity of the start and the stop codons, the open conformation could facilitate even direct loading of the 40S ribosomal subunit to a vicinity of the AUG codon, a mode that is usually prohibited during de novo initiation in eukaryotes [45]. Recently, we demonstrated that the 40S subunit bound to 3’ end translation enhancer of plant virus genomic RNA can be positioned by specific 5’–3’ RNA–RNA interaction on certain AUG codons directly, even downstream of other 5′ proximal AUGs [85]. Because of the diversity of 5’ and 3’ UTRs of cellular mRNAs, such precise positioning of 40S at arbitrary 5’ UTR seems to be unlikely, but the recycled 40S subunit can just bind to the internal part of 5′ UTR proximal to termination site and start non-directional ATP-independent random wandering, seeking for the closest AUG codon. Time necessary to locate AUG by this one-dimensional diffusion should be proportional to the square of nucleotide chain length, which could explain the relatively short optimal 5’ UTR length for efficient CLAR (~80 nt; Figure 4). Alternatively, the decrease of CLAR efficiency with the increase of a 5′ UTR length may reflect the limited efficiency of eIF4F/4A-independent scanning or increased RNA secondary structure stability in the case of long leaders.

Interestingly, it was demonstrated recently that m^6^A methylation of 5′ UTR also promotes cap-independent yet 5′ end-dependent initiation of translation that does not require eIF4F [86]. Moreover, it was shown that such methylation allows initiation even at circular cellular RNAs, i.e., internal initiation [87]. Thus, METTL3-promoted mRNA looping [28] could be a good example of a cellular mechanism where eIF4F is necessary for mRNA circularization but is not required as the eIF4A-mediated locomotive of scanning.

Although we are still far from a full understanding of the mechanisms by which mRNA topology affects translation efficiency, the findings presented here provide strong evidence that CLAR can be the main purpose of closed-loop formation, and the features of this process can be quite different from the canonical initiation pathway.

## 4. Materials and Methods 

### 4.1. Plasmids and In Vitro Transcription 

All DNA templates for the synthesis of *Fluc* mRNAs with rabbit β-globin 5’ UTR were prepared on the basis of the pGL3R-β-glo plasmid [41]. mRNA constructs with the LINE-1 derived 5’ UTRs of different length were based on a set of pGL1 plasmid derivatives, where fragments of the human L1 retrotransposon 5’ UTR were inserted upstream of *Fluc* ORF [49]. Plasmids encoding HSPA1A, MYC and APAF1 5′ UTRs [41,42,88], as well as the PTV and the CrPV IRESs [46,89], were described earlier. PCR products amplified from the corresponding plasmids were used as a template for mRNA synthesis (for the complete list of plasmid/primers combinations used, see Supplementary Data, Table S1). PCR reactions were performed using Expand High Fidelity PCR System kit (Roche Diagnostics, Mannheim, Germany) in accordance with manufacturer recommendations. In vitro transcription was performed according to Pokrovskaya and Gurevich [90] with minor modifications. Reaction mixtures contained 2 mM each ATP, GTP, and CTP, 0.3 mM GTP, 6 mM m^7^GpppG (NEB; except for the IRES containing constructs), and 50 µg/mL of a corresponding PCR product. The resulting mRNAs were purified by phenol extraction, spin gel-filtration, and NH_4_OAc/ethanol precipitation and checked for integrity by MOPS/formaldehyde agarose gel electrophoresis. Internally [^32^P]-radiolabeled transcript was obtained by the addition of 100 µCi [α-^32^P]UTP to the same transcription reaction. 

### 4.2. In Vitro Translation

Whole-cell extracts were prepared from mouse Krebs-2 ascites cells as described by Dmitriev et al. [42]. The final translation mixture contained 50% v/v Krebs-2 extract, 100 µg/mL creatine phosphokinase, 500 U/mL RNase inhibitor, 50 mg/mL calf total tRNA, 25 μM each amino acid, 1 mM ATP, 0.2 mM GTP, and 8 mM creatine phosphate in 20 mM HEPES–KOH buffer pH 7.6 with 0.6 mM Mg(OAc)_2_, 100 mM KOAc, 1 mM DTT, 0.5 mM spermidine and 0.1 mM luciferin. When indicated, the recombinant eIF4A R362Q, obtained as described previously [78], was added to the final concentration of 40 μg/mL. Reaction components were mixed on ice, adjusted to 80% of the final volume, and incubated for 2 min at 30 °C. A quantity of 2 µl of preheated 5-fold concentrated (125 nM) mRNA were diluted with 8 μL of the prepared reaction mixture and immediately put into the temperature-controlled cell of a Chemilum-12 multichannel luminometer. The intensity of light emission generated through luciferase activity was measured continuously by collecting the streaming data in steps of 2.5 s. The kinetic curves were analyzed with Igor Pro 6.0 data processing software (Wavemetrics, Portland, OR). Initial translation rate was determined as a linear approximation of a 5 min fragment of a kinetic curve right after appearance of luciferase activity. Maximum translation rate was determined as a maximal value of the slope of linear approximations obtained for a 5 min window sliding along the whole kinetic curve of 90 min translation reaction with 2.5 s step. 

### 4.3. Sedimentation Analysis of Polyribosomes

The 50 μL Krebs-2 reaction mixtures with [^32^P]-labeled βgloFlucA50 mRNA were collected after 15 and 45 min of translation, chilled on ice, supplemented with cycloheximide up to 0.01 mg/mL, and layered atop a linear 15–45% sucrose gradient in 12 mL Ultra-Clear Beckman tubes containing 25 mM Tris–HCl pH 7.6, 5 mM MgCl2, 100 mM KCl, 0.1 mM EDTA, and 0.01 mg/mL cycloheximide. Samples were subjected to centrifugation for 2 h 45 min in a SW-41 rotor in an Optima L-90K (Beckman-Coulter) ultracentrifuge at 37,000 rpm at 4 °C. Gradients were fractionated starting from the bottom of the tubes, and the radioactivity of 0.5 mL fractions was determined through Cherenkov counting. The same gradient with 20 μL of HEK293T cell lysate loaded was fractionated with continuous measurement of the optical density at 254 nm with UVCord 2238 (Pharmacia Biotech, Uppsala, Sweden). These data are intended to visualize polysome distribution along the gradient, since the absorbance curve of fractionated Krebs-2 system did not allow us to distinguish polysome peaks clearly.

## Supplementary Materials

The following are available online at https://www.mdpi.com/1422-0067/21/5/1677/s1.

## Abbreviations

AR	acceleration rate
CLAR	closed-loop assisted reinitiation
Fluc	firefly luciferase
IRES	internal ribosome entry site
UTR	untranslated region
PTV	porcine teschovirus
CrPV	cricket paralysis virus

## References

[1] Mathias A.P., Williamson R., Huxley H.E., Page S. (1964). Occurrence and Function of Polysomes in Rabbit Reticulocytes. J. Mol. Biol..

[2] Shelton E., Kuff E.L. (1966). Substructure and configuration of ribosomes isolated from mammalian cells. J. Mol. Biol..

[3] Ladhoff A.M., Uerlings I., Rosenthal S. (1981). Electron microscopic evidence of circular molecules in 9-S globin mRNA from rabbit reticulocytes. Mol. Biol. Rep..

[4] Christensen A.K., Kahn L.E., Bourne C.M. (1987). Circular polysomes predominate on the rough endoplasmic reticulum of somatotropes and mammotropes in the rat anterior pituitary. Am. J. Anat..

[5] Philipps G.R. (1965). Haemoglobin Synthesis and Polysomes in Intact Reticulocytes. Nature.

[6] Jacobson A., Hershey J.W.B., Mathews M.B., Sonenberg N. (1996). Poly(A) Metabolism and Translation: The Closed-loop Model. Translational Control.

[7] Jacobson A., Favreau M. (1983). Possible involvement of poly(A) in protein synthesis. Nucleic Acids Res..

[8] Palatnik C.M., Wilkins C., Jacobson A. (1984). Translational control during early Dictyostelium development: Possible involvement of poly(A) sequences. Cell.

[9] Gallie D.R. (1991). The cap and poly(A) tail function synergistically to regulate mRNA translational efficiency. Genes Dev..

[10] Iizuka N., Najita L., Franzusoff A., Sarnow P. (1994). Cap-dependent and cap-independent translation by internal initiation of mRNAs in cell extracts prepared from Saccharomyces cerevisiae. Mol. Cell. Biol..

[11] Tarun S.Z., Sachs A.B. (1995). A common function for mRNA 5′ and 3′ ends in translation initiation in yeast. Genes Dev..

[12] Tarun S.Z., Sachs A.B. (1996). Association of the yeast poly(A) tail binding protein with translation initiation factor eIF-4G. EMBO J..

[13] Wells S.E., Hillner P.E., Vale R.D., Sachs A.B. (1998). Circularization of mRNA by eukaryotic translation initiation factors. Mol. Cell.

[14] Le H., Tanguay R.L., Balasta M.L., Wei C.C., Browning K.S., Metz A.M., Goss D.J., Gallie D.R. (1997). Translation initiation factors eIF-iso4G and eIF-4B interact with the poly(A)-binding protein and increase its RNA binding activity. J. Biol. Chem..

[15] Imataka H., Gradi A., Sonenberg N. (1998). A newly identified N-terminal amino acid sequence of human eIF4G binds poly(A)-binding protein and functions in poly(A)-dependent translation. EMBO J..

[16] Archer S.K., Shirokikh N.E., Hallwirth C.V., Beilharz T.H., Preiss T. (2015). Probing the closed-loop model of mRNA translation in living cells. RNA Biol..

[17] Thompson M.K., Rojas-Duran M.F., Gangaramani P., Gilbert W.V. (2016). The ribosomal protein Asc1/RACK1 is required for efficient translation of short mRNAs. eLife.

[18] Afonina Z.A., Myasnikov A.G., Shirokov V.A., Klaholz B.P., Spirin A.S. (2014). Formation of circular polyribosomes on eukaryotic mRNA without cap-structure and poly(A)-tail: A cryo electron tomography study. Nucleic Acids Res..

[19] Neusiedler J., Mocquet V., Limousin T., Ohlmann T., Morris C., Jalinot P. (2012). INT6 interacts with MIF4GD/SLIP1 and is necessary for efficient histone mRNA translation. RNA.

[20] von Moeller H., Lerner R., Ricciardi A., Basquin C., Marzluff W.F., Conti E. (2013). Structural and biochemical studies of SLIP1-SLBP identify DBP5 and eIF3g as SLIP1-binding proteins. Nucleic Acids Res..

[21] Vende P., Piron M., Castagne N., Poncet D. (2000). Efficient translation of rotavirus mRNA requires simultaneous interaction of NSP3 with the eukaryotic translation initiation factor eIF4G and the mRNA 3′ end. J. Virol..

[22] Groft C.M., Burley S.K. (2002). Recognition of eIF4G by rotavirus NSP3 reveals a basis for mRNA circularization. Mol. Cell.

[23] Gratia M., Sarot E., Vende P., Charpilienne A., Baron C.H., Duarte M., Pyronnet S., Poncet D. (2015). Rotavirus NSP3 Is a Translational Surrogate of the Poly(A) Binding Protein-Poly(A) Complex. J. Virol..

[24] Bergamini G., Preiss T., Hentze M.W. (2000). Picornavirus IRESes and the poly(A) tail jointly promote cap-independent translation in a mammalian cell-free system. RNA.

[25] Michel Y.M., Borman A.M., Paulous S., Kean K.M. (2001). Eukaryotic initiation factor 4G-poly(A) binding protein interaction is required for poly(A) tail-mediated stimulation of picornavirus internal ribosome entry segment-driven translation but not for X-mediated stimulation of hepatitis C virus translation. Mol. Cell. Biol..

[26] Svitkin Y.V., Imataka H., Khaleghpour K., Kahvejian A., Liebig H.D., Sonenberg N. (2001). Poly(A)-binding protein interaction with elF4G stimulates picornavirus IRES-dependent translation. RNA.

[27] Ito T., Lai M.M. (1999). An internal polypyrimidine-tract-binding protein-binding site in the hepatitis C virus RNA attenuates translation, which is relieved by the 3′-untranslated sequence. Virology.

[28] Choe J., Lin S., Zhang W., Liu Q., Wang L., Ramirez-Moya J., Du P., Kim W., Tang S., Sliz P. (2018). mRNA circularization by METTL3-eIF3h enhances translation and promotes oncogenesis. Nature.

[29] Wang X., Zhao B.S., Roundtree I.A., Lu Z., Han D., Ma H., Weng X., Chen K., Shi H., He C. (2015). N(6)-methyladenosine Modulates Messenger RNA Translation Efficiency. Cell.

[30] Thompson M.K., Gilbert W.V. (2017). mRNA length-sensing in eukaryotic translation: Reconsidering the “closed loop” and its implications for translational control. Curr. Genet..

[31] Fakim H., Fabian M.R. (2019). Communication Is Key: 5′-3′ Interactions that Regulate mRNA Translation and Turnover. Adv. Exp. Med. Biol..

[32] Vicens Q., Kieft J.S., Rissland O.S. (2018). Revisiting the Closed-Loop Model and the Nature of mRNA 5′-3′ Communication. Mol. Cell.

[33] Pelletier J., Sonenberg N. (2019). The Organizing Principles of Eukaryotic Ribosome Recruitment. Annu. Rev. BioChem..

[34] Madin K., Sawasaki T., Kamura N., Takai K., Ogasawara T., Yazaki K., Takei T., Miura K., Endo Y. (2004). Formation of circular polyribosomes in wheat germ cell-free protein synthesis system. FEBS Lett..

[35] Mayr C. (2017). Regulation by 3′-Untranslated Regions. Annu. Rev. Genet..

[36] Rogers D.W., Bottcher M.A., Traulsen A., Greig D. (2017). Ribosome reinitiation can explain length-dependent translation of messenger RNA. PLoS Comput. Biol..

[37] Marshall E., Stansfield I., Romano M.C. (2014). Ribosome recycling induces optimal translation rate at low ribosomal availability. J. R. Soc. Interface.

[38] Kolb V.A., Makeyev E.V., Spirin A.S. (1994). Folding of firefly luciferase during translation in a cell-free system. EMBO J..

[39] Alekhina O.M., Vassilenko K.S., Spirin A.S. (2007). Translation of non-capped mRNAs in a eukaryotic cell-free system: Acceleration of initiation rate in the course of polysome formation. Nucleic Acids Res..

[40] Vassilenko K.S., Alekhina O.M., Dmitriev S.E., Shatsky I.N., Spirin A.S. (2011). Unidirectional constant rate motion of the ribosomal scanning particle during eukaryotic translation initiation. Nucleic Acids Res..

[41] Andreev D.E., Dmitriev S.E., Terenin I.M., Prassolov V.S., Merrick W.C., Shatsky I.N. (2009). Differential contribution of the m7G-cap to the 5′ end-dependent translation initiation of mammalian mRNAs. Nucleic Acids Res..

[42] Dmitriev S.E., Andreev D.E., Adyanova Z.V., Terenin I.M., Shatsky I.N. (2009). Efficient cap-dependent translation of mammalian mRNAs with long and highly structured 5′-untranslated regions in vitro and in vivo. Mol. Biol. (Mosk.).

[43] Shatsky I.N., Dmitriev S.E., Andreev D.E., Terenin I.M. (2014). Transcriptome-wide studies uncover the diversity of modes of mRNA recruitment to eukaryotic ribosomes. Crit. Rev. BioChem. Mol. Biol..

[44] Shatsky I.N., Dmitriev S.E., Terenin I.M., Andreev D.E. (2010). Cap- and IRES-independent scanning mechanism of translation initiation as an alternative to the concept of cellular IRESs. Mol. Cells.

[45] Terenin I.M., Smirnova V.V., Andreev D.E., Dmitriev S.E., Shatsky I.N. (2017). A researcher’s guide to the galaxy of IRESs. Cell. Mol. Life Sci. CMLS.

[46] Pisarev A.V., Chard L.S., Kaku Y., Johns H.L., Shatsky I.N., Belsham G.J. (2004). Functional and structural similarities between the internal ribosome entry sites of hepatitis C virus and porcine teschovirus, a picornavirus. J. Virol..

[47] Wilson J.E., Pestova T.V., Hellen C.U., Sarnow P. (2000). Initiation of protein synthesis from the A site of the ribosome. Cell.

[48] Sorensen M.A., Pedersen S. (1998). Determination of the peptide elongation rate in vivo. Methods Mol. Biol..

[49] Dmitriev S.E., Andreev D.E., Terenin I.M., Olovnikov I.A., Prassolov V.S., Merrick W.C., Shatsky I.N. (2007). Efficient translation initiation directed by the 900-nucleotide-long and GC-rich 5′ untranslated region of the human retrotransposon LINE-1 mRNA is strictly cap dependent rather than internal ribosome entry site mediated. Mol. Cell. Biol..

[50] Leppek K., Das R., Barna M. (2018). Functional 5′ UTR mRNA structures in eukaryotic translation regulation and how to find them. Nat. Rev. Mol. Cell Biol..

[51] Akulich K.A., Andreev D.E., Terenin I.M., Smirnova V.V., Anisimova A.S., Makeeva D.S., Arkhipova V.I., Stolboushkina E.A., Garber M.B., Prokofjeva M.M. (2016). Four translation initiation pathways employed by the leaderless mRNA in eukaryotes. Sci. Rep..

[52] Tian B., Hu J., Zhang H., Lutz C.S. (2005). A large-scale analysis of mRNA polyadenylation of human and mouse genes. Nucleic Acids Res..

[53] Nicholson A.L., Pasquinelli A.E. (2019). Tales of Detailed Poly(A) Tails. Trends Cell Biol..

[54] Weill L., Belloc E., Bava F.A., Mendez R. (2012). Translational control by changes in poly(A) tail length: Recycling mRNAs. Nat. Struct. Mol. Biol..

[55] Svitkin Y.V., Sonenberg N. (2004). An efficient system for cap- and poly(A)-dependent translation in vitro. Methods Mol. Biol..

[56] Eliseeva I.A., Lyabin D.N., Ovchinnikov L.P. (2013). Poly(A)-binding proteins: Structure, domain organization, and activity regulation. Biochem. Biokhimiia.

[57] Pause A., Methot N., Svitkin Y., Merrick W.C., Sonenberg N. (1994). Dominant negative mutants of mammalian translation initiation factor eIF-4A define a critical role for eIF-4F in cap-dependent and cap-independent initiation of translation. EMBO J..

[58] Pause A., Methot N., Sonenberg N. (1993). The HRIGRXXR region of the DEAD box RNA helicase eukaryotic translation initiation factor 4A is required for RNA binding and ATP hydrolysis. Mol. Cell. Biol..

[59] Pestova T.V., Kolupaeva V.G. (2002). The roles of individual eukaryotic translation initiation factors in ribosomal scanning and initiation codon selection. Genes Dev..

[60] Villalba A., Coll O., Gebauer F. (2011). Cytoplasmic polyadenylation and translational control. Curr. Opin. Genet. Dev..

[61] Machida K., Shigeta T., Yamamoto Y., Ito T., Svitkin Y., Sonenberg N., Imataka H. (2018). Dynamic interaction of poly(A)-binding protein with the ribosome. Sci. Rep..

[62] Kahvejian A., Svitkin Y.V., Sukarieh R., M’Boutchou M.N., Sonenberg N. (2005). Mammalian poly(A)-binding protein is a eukaryotic translation initiation factor, which acts via multiple mechanisms. Genes Dev..

[63] Borman A.M., Michel Y.M., Kean K.M. (2000). Biochemical characterisation of cap-poly(A) synergy in rabbit reticulocyte lysates: The eIF4G-PABP interaction increases the functional affinity of eIF4E for the capped mRNA 5′-end. Nucleic Acids Res..

[64] Bushell M., Wood W., Carpenter G., Pain V.M., Morley S.J., Clemens M.J. (2001). Disruption of the interaction of mammalian protein synthesis eukaryotic initiation factor 4B with the poly(A)-binding protein by caspase- and viral protease-mediated cleavages. J. Biol. Chem..

[65] Wei C.C., Balasta M.L., Ren J., Goss D.J. (1998). Wheat germ poly(A) binding protein enhances the binding affinity of eukaryotic initiation factor 4F and (iso)4F for cap analogues. Biochemistry.

[66] Bi X., Goss D.J. (2000). Wheat germ poly(A)-binding protein increases the ATPase and the RNA helicase activity of translation initiation factors eIF4A, eIF4B, and eIF-iso4F. J. Biol. Chem..

[67] Searfoss A., Dever T.E., Wickner R. (2001). Linking the 3′ poly(A) tail to the subunit joining step of translation initiation: Relations of Pab1p, eukaryotic translation initiation factor 5b (Fun12p), and Ski2p-Slh1p. Mol. Cell. Biol..

[68] Filbin M.E., Kieft J.S. (2016). Linking Alpha to Omega: Diverse and dynamic RNA-based mechanisms to regulate gene expression by 5′-to-3′ communication. F1000Research.

[69] Adivarahan S., Livingston N., Nicholson B., Rahman S., Wu B., Rissland O.S., Zenklusen D. (2018). Spatial Organization of Single mRNPs at Different Stages of the Gene Expression Pathway. Mol. Cell.

[70] Khong A., Parker R. (2018). mRNP architecture in translating and stress conditions reveals an ordered pathway of mRNP compaction. J. Cell Biol..

[71] Metkar M., Ozadam H., Lajoie B.R., Imakaev M., Mirny L.A., Dekker J., Moore M.J. (2018). Higher-Order Organization Principles of Pre-translational mRNPs. Mol. Cell.

[72] Kopeina G.S., Afonina Z.A., Gromova K.V., Shirokov V.A., Vasiliev V.D., Spirin A.S. (2008). Step-wise formation of eukaryotic double-row polyribosomes and circular translation of polysomal mRNA. Nucleic Acids Res..

[73] Afonina Z.A., Myasnikov A.G., Shirokov V.A., Klaholz B.P., Spirin A.S. (2015). Conformation transitions of eukaryotic polyribosomes during multi-round translation. Nucleic Acids Res..

[74] Pierron G., Weil D. (2018). Re-viewing the 3D Organization of mRNPs. Mol. Cell.

[75] Lai W.C., Kayedkhordeh M., Cornell E.V., Farah E., Bellaousov S., Rietmeijer R., Salsi E., Mathews D.H., Ermolenko D.N. (2018). mRNAs and lncRNAs intrinsically form secondary structures with short end-to-end distances. Nat. Commun..

[76] Hoshino S., Imai M., Kobayashi T., Uchida N., Katada T. (1999). The eukaryotic polypeptide chain releasing factor (eRF3/GSPT) carrying the translation termination signal to the 3′-Poly(A) tail of mRNA. Direct association of erf3/GSPT with polyadenylate-binding protein. J. Biol. Chem..

[77] Pestova T.V., Shatsky I.N., Fletcher S.P., Jackson R.J., Hellen C.U. (1998). A prokaryotic-like mode of cytoplasmic eukaryotic ribosome binding to the initiation codon during internal translation initiation of hepatitis C and classical swine fever virus RNAs. Genes Dev..

[78] Terenin I.M., Dmitriev S.E., Andreev D.E., Royall E., Belsham G.J., Roberts L.O., Shatsky I.N. (2005). A cross-kingdom internal ribosome entry site reveals a simplified mode of internal ribosome entry. Mol. Cell. Biol..

[79] Agalarov S., Sakharov P.A., Fattakhova D., Sogorin E.A., Spirin A.S. (2014). Internal translation initiation and eIF4F/ATP-independent scanning of mRNA by eukaryotic ribosomal particles. Sci. Rep..

[80] Mancera-Martinez E., Brito Querido J., Valasek L.S., Simonetti A., Hashem Y. (2017). ABCE1: A special factor that orchestrates translation at the crossroad between recycling and initiation. RNA Biol..

[81] Valasek L.S., Zeman J., Wagner S., Beznoskova P., Pavlikova Z., Mohammad M.P., Hronova V., Herrmannova A., Hashem Y., Gunisova S. (2017). Embraced by eIF3: Structural and functional insights into the roles of eIF3 across the translation cycle. Nucleic Acids Res..

[82] Ivanov A., Mikhailova T., Eliseev B., Yeramala L., Sokolova E., Susorov D., Shuvalov A., Schaffitzel C., Alkalaeva E. (2016). PABP enhances release factor recruitment and stop codon recognition during translation termination. Nucleic Acids Res..

[83] Schleich S., Strassburger K., Janiesch P.C., Koledachkina T., Miller K.K., Haneke K., Cheng Y.S., Kuechler K., Stoecklin G., Duncan K.E. (2014). DENR-MCT-1 promotes translation re-initiation downstream of uORFs to control tissue growth. Nature.

[84] Young D.J., Makeeva D.S., Zhang F., Anisimova A.S., Stolboushkina E.A., Ghobakhlou F., Shatsky I.N., Dmitriev S.E., Hinnebusch A.G., Guydosh N.R. (2018). Tma64/eIF2D, Tma20/MCT-1, and Tma22/DENR Recycle Post-termination 40S Subunits In Vivo. Mol. Cell.

[85] Gao F., Alekhina O.M., Vassilenko K.S., Simon A.E. (2018). Unusual dicistronic expression from closely spaced initiation codons in an umbravirus subgenomic RNA. Nucleic Acids Res..

[86] Meyer K.D., Patil D.P., Zhou J., Zinoviev A., Skabkin M.A., Elemento O., Pestova T.V., Qian S.B., Jaffrey S.R. (2015). 5′ UTR m(6)A Promotes Cap-Independent Translation. Cell.

[87] Yang Y., Fan X., Mao M., Song X., Wu P., Zhang Y., Jin Y., Yang Y., Chen L.L., Wang Y. (2017). Extensive translation of circular RNAs driven by N(6)-methyladenosine. Cell Res..

[88] Rubtsova M.P., Sizova D.V., Dmitriev S.E., Ivanov D.S., Prassolov V.S., Shatsky I.N. (2003). Distinctive properties of the 5′-untranslated region of human hsp70 mRNA. J. Biol. Chem..

[89] Prokhorova I.V., Akulich K.A., Makeeva D.S., Osterman I.A., Skvortsov D.A., Sergiev P.V., Dontsova O.A., Yusupova G., Yusupov M.M., Dmitriev S.E. (2016). Amicoumacin A induces cancer cell death by targeting the eukaryotic ribosome. Sci. Rep..

[90] Pokrovskaya I.D., Gurevich V.V. (1994). In vitro transcription: Preparative RNA yields in analytical scale reactions. Anal. BioChem..

