# Supplementary material for: Functional Cyclization of Eukaryotic mRNAs
Source: Int J Mol Sci. 2020 Feb 29;21(5):1677. doi: 10.3390/ijms21051677 (PMC7084953; doi:10.3390/ijms21051677)
Supplement: Supplementary file 1 [file ijms-21-01677-s001.zip › Supplementary materials.pdf]

International Journal of
Molecular Sciences

MDPI

Functional cyclization of eukaryotic mRNAs

Olga M. Alekhina, Ilya M. Terenin, Alexander S. Spirin,

Sergey E. Dmitriev, and Konstantin S. Vassilenko

Supplementary materials

Table S1. Plasmids and primers for the amplification of PCR products used as transcription templates for mRNA synthesis.

mRNA construct	Plasmid	Forward primer	Reverse primer
βgloFlucA50	pGL3R-β-glo [1]	CGCCGTAATACGACTCACTATAGGGACACTTGCTTTTGACACAACTGTG	$(T)_{50}$AACTTGTTTATTGCAGCTTATAATGG
βgloFluc	– 〃 –	– 〃 –	AACTTGTTTATTGCAGCTTATAATGG
βgloFlucA15	– 〃 –	– 〃 –	$(T)_{15}$AACTTGTTTATTGCAGCTTATAATGG
βgloFlucA27	– 〃 –	– 〃 –	$(T)_{27}$AACTTGTTTATTGCAGCTTATAATGG
βgloFlucA98	– 〃 –	– 〃 –	$(T)_{98}$AACTTGTTTATTGCAGCTTATAATGG
βgloFluc15A50	– 〃 –	– 〃 –	$(T)_{50}$GACTCTAGAATTACACGGCGATC
βgloFluc30A50	– 〃 –	– 〃 –	$(T)_{50}$ATGTATCTTATCATGTCTGCTCGA
βgloFluc300A50	– 〃 –	– 〃 –	$(T)_{50}$CAGTTCCGCCCATTCTCCGCT
βgloFluc1000A50	– 〃 –	– 〃 –	$(T)_{50}$GAAGGGAGAAAGGCGGACAGG
5FlucA50	– 〃 –	CGCCGTAATACGACTCACTATAGGAGAATGGCCACGGAAGACG	$(T)_{50}$AACTTGTTTATTGCAGCTTATAATGG
L192FlucA50	pRluc-L1Δ(133-887)-Fluc [2]	CGCCGTAATACGACTCACTATAGGGAGCTTATCGATACCGTCG	– 〃 –
L280FlucA50	pRluc-L1Δ(1-665)-Fluc [2]	– 〃 –	– 〃 –
L555FlucA50	pRluc-L1Δ(103-386)-Fluc [2]	– 〃 –	– 〃 –
L948FlucA50	pRluc-L15'UTR-Fluc [2]	– 〃 –	– 〃 –

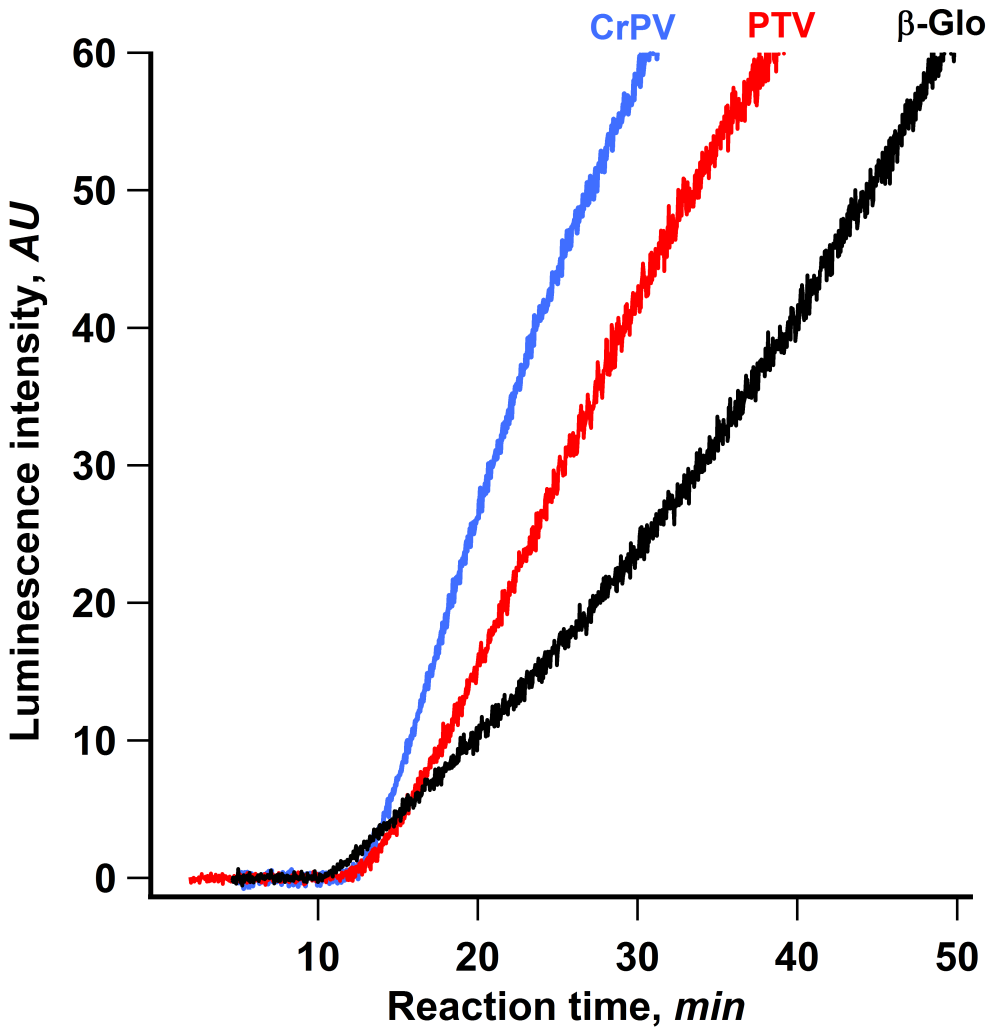

Figure S1. IRES-containing uncapped polyadenylated transcripts with no scanning reveal no acceleration of translation. Continuous in situ monitoring of the translation of PTV-Fluc [3] and CrPV-Fluc [4] transcripts in a Krebs-2 cell-free system. Kinetic curve of βgloFlucA50 mRNA translation added as a control.

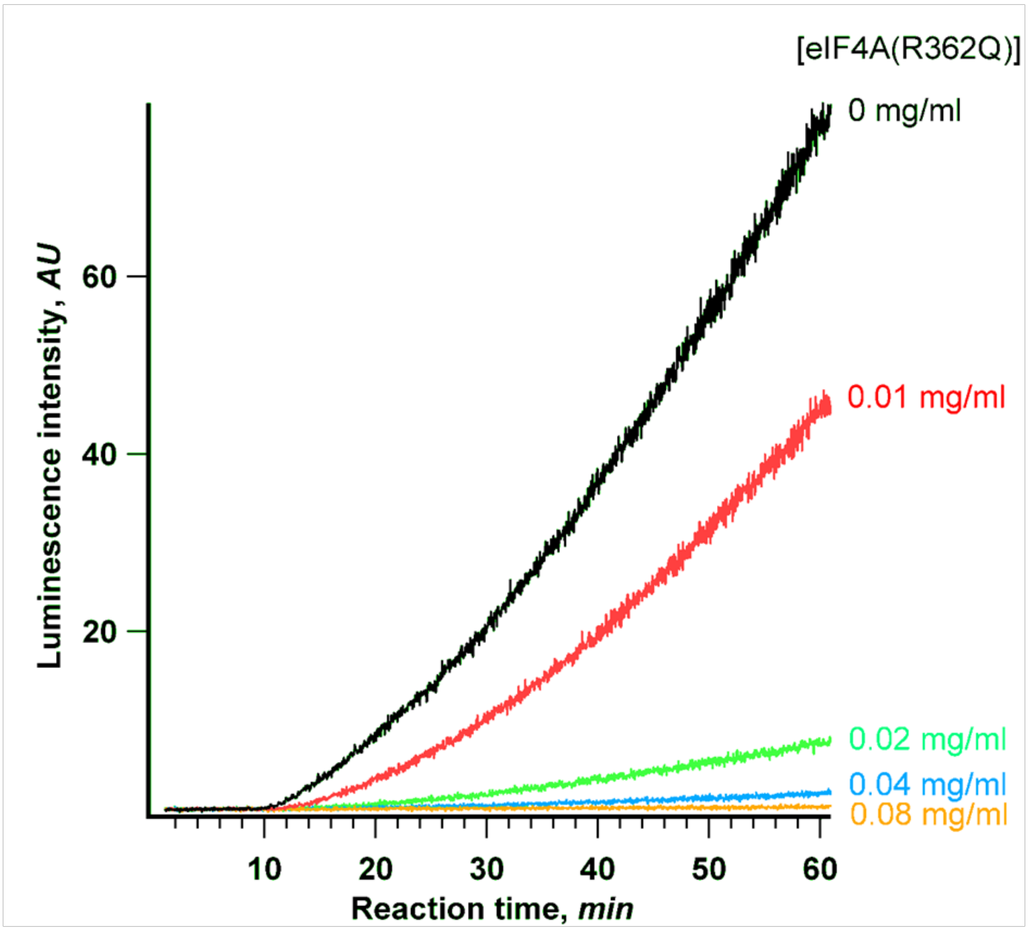

Figure S2. Inhibitory effect of the addition of eIF4A R362Q mutant to Krebs-2 *in vitro* system translating 25 nM of a luciferase βgloFlucA50 mRNA. The indicated values denote the final concentration of eIF4A(R362Q) in the corresponding reaction mixture.

References

1. Andreev, D.E.; Dmitriev, S.E.; Terenin, I.M.; Prassolov, V.S.; Merrick,W.C.; Shatsky, I.N. Differential contribution of the m7G-cap to the 5′ end-dependent translation initiation of mammalian mRNAs. *Nucleic Acids Res.* **2009**, *37*, 6135-6147.
2. Dmitriev, S.E.; Andreev, D.E.; Terenin, I.M.; Olovnikov, I.A.; Prassolov, V.S.; Merrick, W.C.; Shatsky, I.N. Efficient translation initiation directed by the 900-nucleotide-long and GC-rich 5′ untranslated region of the human retrotransposon LINE-1 mRNA is strictly cap dependent rather than internal ribosome entry site mediated. *Mol. Cell. Biol.* **2007**, *27*, 4685-4697.
3. Pisarev, A.V.; Chard, L.S.; Kaku, Y.; Johns, H.L.; Shatsky, I.N.; Belsham, G.J. Functional and structural similarities between the internal ribosome entry sites of hepatitis C virus and porcine teschovirus, a picornavirus. *Journal of virology* **2004**, *78*, 4487-97.
4. Prokhorova, I.V.; Akulich, K.A.; Makeeva, D.S.; Osterman, I.A.; Skvortsov, D.A.; Sergiev, P.V.; Dontsova, O.A.; Yusupova, G.; Yusupov, M.M.; Dmitriev, S.E. Amicoumacin A induces cancer cell death by targeting the eukaryotic ribosome. Scientific reports **2016**, *6*, 27720.
